# Multidisciplinary Management and Staged Surgical Treatment for Chronic Thromboembolic Pulmonary Hypertension and Subclavian Vein Thrombosis Caused by Venous Thoracic Outlet Syndrome

**DOI:** 10.7759/cureus.84953

**Published:** 2025-05-28

**Authors:** Muhammad Zeeshan, Gregory J Pearl, Murali M Chakinala, Kunal D Kotkar, Morey A Blinder, Pavan K Kavali, Michael M Madani, Robert W Thompson

**Affiliations:** 1 Center for Thoracic Outlet Syndrome and Division of Vascular Surgery, Department of Surgery, Washington University School of Medicine, St. Louis, USA; 2 Division of Vascular Surgery, Department of Surgery, Baylor University Medical Center and Baylor Scott and White Heart and Vascular Hospital, Dallas, USA; 3 Division of Pulmonary and Critical Care Medicine, Department of Medicine, Washington University School of Medicine, St. Louis, USA; 4 Section of Cardiac Surgery, Department of Surgery, Washington University School of Medicine, St. Louis, USA; 5 Division of Hematology, Department of Medicine, Washington University School of Medicine, St. Louis, MO, USA; 6 Section of Interventional Radiology, Department of Radiology and Mallinckrodt Institute of Radiology, Washington University School of Medicine, St. Louis, USA; 7 Division of Cardiovascular and Thoracic Surgery, Department of Surgery, University of California San Diego, San Diego, USA

**Keywords:** deep vein thrombosis, medical management, pulmonary embolism, pulmonary hypertension, surgical treatment, upper extremity

## Abstract

Chronic thromboembolic pulmonary hypertension (CTEPH) is an uncommon complication of deep vein thrombosis (DVT) and pulmonary embolism (PE), occurring in 3-5% of patients despite therapeutic anticoagulation. Venous thoracic outlet syndrome (VTOS) causing subclavian vein (SCV) thrombosis is also an uncommon condition, not frequently associated with clinically significant PE. In this report, we present two patients with CTEPH and SCV thrombosis caused by VTOS who had successful multidisciplinary management and staged surgical treatment for both conditions. Each patient was young and otherwise healthy before presenting with progressively worsening shortness of breath. Both were found to have multiple peripheral PE and features of respiratory failure characteristic of CTEPH, along with axillary-SCV thrombosis and no other source of DVT. Each patient was treated with anticoagulation and riociguat but had ongoing pulmonary symptoms, and staged surgical treatment was recommended. The first stage was conducted by bilateral pulmonary thromboendarterectomy performed under deep hypothermic circulatory arrest. The second stage was conducted several months later after resolution of pulmonary hypertension, by paraclavicular thoracic outlet decompression that included complete first rib resection with a patent axillary-SCV on subsequent upper extremity imaging. Each patient recovered well with resolution of respiratory and upper extremity symptoms and a return to unrestricted activity by three months after the second operation. These patients are some of the first to have successful staged surgical treatment for CTEPH and SCV thrombosis caused by VTOS, illustrating the value of comprehensive multidisciplinary management for this unusual combination of rare conditions.

## Introduction

Chronic thromboembolic pulmonary hypertension (CTEPH) is an uncommon complication of deep vein thrombosis (DVT) and pulmonary embolism (PE) that can cause progressive respiratory failure, occurring in 3-5% of patients despite therapeutic anticoagulation [1-9]. Venous thoracic outlet syndrome (VTOS) with subclavian vein (SCV) thrombosis (Paget-Schroetter syndrome; "effort thrombosis") is also an uncommon condition, which may lead to upper extremity disability and post-thrombotic syndrome, but it is not frequently associated with clinically significant PE [10-19]. In this report, we present two patients with CTEPH and SCV thrombosis caused by VTOS with no other source of DVT, with successful multidisciplinary management and staged surgical treatment for both conditions.

## Case presentation

Case 1

**Figure 1:**
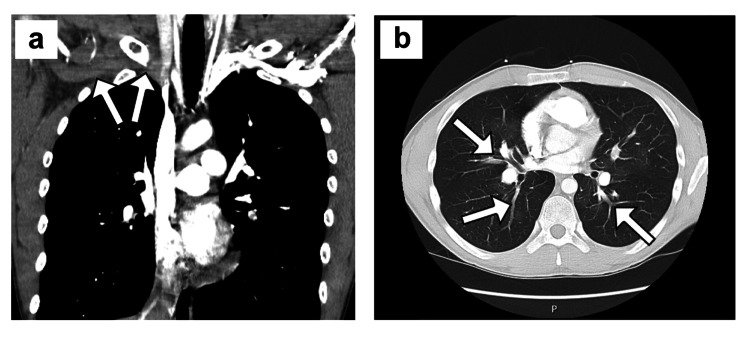
Initial imaging in case 1 a) Coronal view of chest CTA showing right axillary-SCV thrombosis (white arrows). b) Cross-sectional view of chest CTA showing segmental pulmonary artery branch thromboembolism (white arrows). CTA: computed tomography angiography; SCV: subclavian vein

An otherwise healthy 23-year-old man developed cough, shortness of breath, and chest pain in July 2023 and was treated with oral antibiotics for suspected pneumonia. One month later, he presented to a community hospital with increased shortness of breath and spontaneous right upper extremity swelling and cyanotic discoloration. An upper extremity Duplex ultrasound was negative for DVT, but a chest computed tomography angiogram (CTA) showed bilateral PE and he was started on apixaban anticoagulation. In December 2023, the patient had increased shortness of breath and chest pain and again presented to a local emergency room. At that time, a Duplex ultrasound showed right axillary-SCV thrombosis that was confirmed by chest CTA, along with the presence of increased bilateral PE (Figure 1a, 1b). 

The patient was started on intravenous heparin and hospitalized with hypoxic respiratory failure. A transthoracic echocardiogram revealed no central pulmonary artery (PA) embolism, but there was a moderate decrease in right ventricular systolic function, mild to moderate tricuspid regurgitation, and severe pulmonary hypertension with a right ventricular systolic pressure (RVSP) of 90 mm Hg. He had laboratory coagulation studies showing no evidence for a thrombophilia disorder (Table 1) and was maintained on apixaban anticoagulation. 

**Table 1:** Coagulation and autoimmune testing in case 1. Laboratory data for coagulation and autoimmune testing at the time of initial presentation, with no discernable abnormalities. Lupus anticoagulant was not tested due to concomitant treatment with anticoagulation which interferes with the assay. APCR: activated protein C resistance; DNA: deoxyribonucleic acid; IgG: immunoglobin G; IgM: immunoglobin M; IU: international units

	Patient value	Normal reference
Factor V Leiden (APCR)	3.1 ratio	2.3-100 ratio
Protein C activity	79%	60-150%
Protein S antigen, free	132%	55-150%
Antithrombin III activity	109%	80-125%
Cardiolipin antibody, IgG	<1.6 GPL U/mL	<20 GPL U/mL
Cardiolipin antibody, IgM	0.2 MPL U/mL	<20 MPL U/mL
Beta 2 glycoprotein IgG	<1.4 U/mL	<20 U/mL
Beta 2 glycoprotein IgM	0.2 U/mL	<20 U/mL
Anti-double stranded DNA	<1.0 IU/mL	<4.0 IU/mL
Rheumatoid factor	<10.0 IU/mL	0.1-15.0 IU/mL

The patient underwent a right upper extremity venogram with catheter-directed mechanical thrombectomy and balloon angioplasty to restore a patent axillary-SCV. A pulmonary angiogram was then performed with an attempt at mechanical thrombectomy, but only a small amount of subacute clot was retrieved from the left PA and no significant clot from the right PA. The post-procedural PA pressure was 82/33 (mean 50) mmHg despite PA balloon angioplasty.

**Figure 2:**
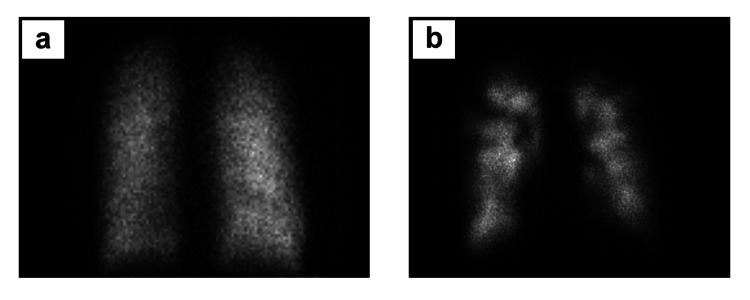
Nuclear medicine evaluation in case 1 a) Xe-133 ventilation images showing a uniform distribution of activity on single-breath and wash-in images and no abnormal Xe-133 retention during the washout phase. b) Intravenous Tc-99m macro-aggregated albumin perfusion images demonstrate multiple mismatched moderate-sized and large segmental perfusion defects in all lobes of both lungs with additional scattered small defects. The discrepancy between scan images demonstrates diminished lung perfusion due to bilateral peripheral pulmonary thromboembolism.

The patient was transferred to an academic medical center on December 29, 2023, where on admission, he had redeveloped swelling and cyanotic discoloration of the entire right arm and dyspnea at rest that required supplemental oxygen (pulse oximetry desaturation to 80% during mild exertion). The 11-item Disabilities of the Arm, Shoulder, and Hand (QuickDASH) score was 48, indicating a moderate level of upper extremity disability [20]. Ventilation-perfusion scintigraphy confirmed severe bilateral perfusion mismatches (Figure 2a, 2b).

**Figure 3:**
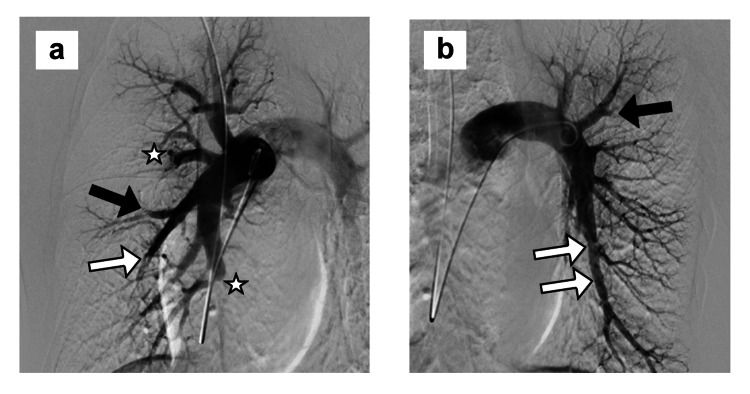
Pulmonary angiography in case 1 PA contrast injections on the a) right and b) left sides, showing changes of chronic thromboembolic disease, including eccentric luminal narrowing (white arrows), rapid tapering of peripheral vessels (black arrows), abrupt cut-offs (white stars) and absence of capillary blushes in multiple regions (not shown in these images), characteristic of CTEPH. CTEPH: chronic thromboembolic pulmonary hypertension; PA: pulmonary artery

A transthoracic echocardiogram showed moderate right ventricular dilatation and an RVSP of 50 mm Hg. Pulmonary arteriography confirmed changes of chronic thromboembolic disease including eccentric narrowing of vessels, abrupt cut-offs, and reduced capillary blushes (Figure 3a, 3b). 

Right heart catheterization showed pre-capillary pulmonary hypertension with PA pressures of 73/26 (mean 40) mmHg, pulmonary artery wedge pressure (PAWP) of 11 mm Hg, and an elevated pulmonary vascular resistance (PVR) of 6.44 Wood units (normal up to 2.0), characteristic of CTEPH (Table 2). 

**Table 2:** Right heart catheterization data in case 1. Catheterization data showing significant pulmonary hypertension (pulmonary vascular resistance > 2 Wood units), with significant improvement at follow-up catheterization two months after pulmonary thromboendarterectomy.

	Initial presentation	Postoperative follow-up
Date of study	01/04/2024	08/12/2024
Systemic artery pressure (mean)	137/85 (102) mmHg	127/81 (97) mmHg
Right atrial pressure	3 mmHg	2 mmHg
Right ventricular pressure (mean)	73/6 (28) mmHg	43/3 (16) mmHg
Pulmonary artery pressure (mean)	73/26 (40) mmHg	43/14 (24) mmHg
Pulmonary artery wedge pressure	11 mmHg	8 mmHg
Arterial oxygen saturation, %	95%	100%
Transpulmonary pressure gradient	29 mmHg	16 mmHg
Diastolic pressure gradient	15 mmHg	6 mmHg
Pulmonary vascular resistance	6.4 Wood units	2.7 Wood units

**Figure 4:**
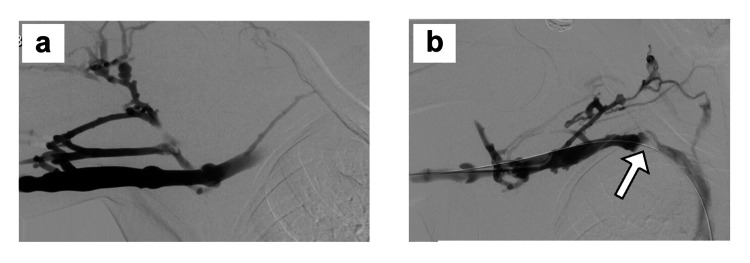
Initial upper extremity venography in case 1 a) Initial right upper extremity venogram showing axillary-SCV thrombosis. b) Venographic image after catheter-directed suction thrombectomy, thrombolysis, and balloon angioplasty for SCV stenosis, showing a patent axillary-SCV with persistent collateral vein filling and residual stenosis in the central SCV (white arrow), characteristic of venous TOS. SCV: subclavian vein; TOS: thoracic outlet syndrome

The pro B-type natriuretic protein level was 780 pg/mL (normal < 300 pg/mL), consistent with a right heart strain. The patient was started on riociguat (a soluble guanylate cyclase stimulator), and he had a gradual reduction in the need for supplemental oxygen. He then underwent right upper extremity catheter venography showing axillary-SCV re-thrombosis, with mechanical thrombectomy and balloon angioplasty up to 12 mm diameter using a drug-coated balloon to recanalize the SCV (Figure 4a, 4b). 

**Figure 5:**
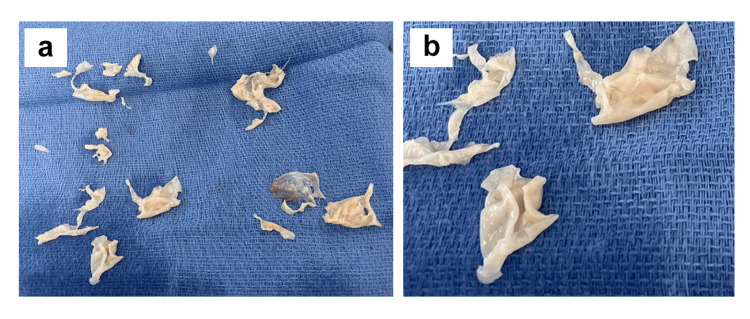
Surgical specimens in case 1 a) Operative photograph of specimens removed at the time of pulmonary thromboendarterectomy and b) close-up view of the same specimens.

The patient had a subsequent reduction in right arm swelling and was maintained on apixaban anticoagulation and riociguat for discharge home. Over the next three months, the patient had mild arm swelling but continued to have dyspnea and persistent physical activity limitations (NYHA class III), and in follow-up office visits, it was recommended that he proceed with staged sequential surgical treatment. On June 4, 2024, he underwent bilateral pulmonary thromboendarterectomy under deep hypothermic circulatory arrest, with an aortic cross-clamp time of 109 minutes, a cardiopulmonary bypass time of 204 minutes, and total circulatory arrest time of 51 minutes. The endarterectomy plane in the pulmonary artery was carried into each of the segmental and subsegmental branches on both right and left sides, representing level III disease in the University of California San Diego classification (Figure 5a, 5b) [7,21,22]. Microscopic examination of the resected surgical specimens demonstrated endothelial tissue with multiple vascular channels, consistent with recanalized organized thrombus. 

The patient had an uncomplicated recovery from surgery with a postoperative pro B-type natriuretic protein level of 134 pg/mL, consistent with the resolution of the right heart strain, and he was discharged home on postoperative day six. Riociguat was discontinued and cardiac catheterization two months later showed significant improvement in hemodynamics with PA pressures 43/14 (mean 24) mmHg, PAWP of 8 mm Hg, and PVR of 2.7 Wood units (Table 2). 

**Figure 6:**
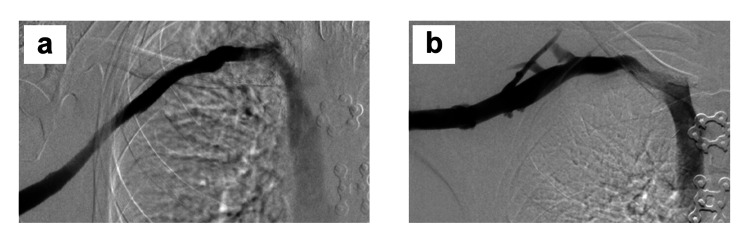
Follow-up venography in case 1 a) Intraoperative completion right upper extremity venogram performed immediately after paraclavicular decompression with complete first rib resection and external venolysis of the axillary-SCV, demonstrating a widely patent axillary-SCV with no filling of collateral veins, and b) right upper extremity venogram performed three months after surgical treatment for venous TOS, showing a widely patent axillary-SCV with rapid flow to the innominate vein. SCV: subclavian vein; TOS: thoracic outlet syndrome

The patient returned to the hospital on September 10, 2024, and underwent successful right paraclavicular thoracic outlet decompression with resection of the anterior and middle scalene muscles, the subclavius muscle, and the entire first rib to the level of the sternum [23]. Following external venolysis to remove fibrous scar tissue from around the axillary-SCV, an intraoperative venogram showed a widely patent SCV with no thrombus or residual stenosis, precluding the need for endovascular intervention or direct SCV reconstruction (Figure 6a). 

The patient recovered well from this operation with no further arm swelling, and he was discharged home with apixaban anticoagulation on postoperative day four. He started physical therapy one month after surgery with a QuickDASH score of 25. At three months after surgery, he had progressively increased activity with a QuickDASH score of 18, and the axillary-SCV was widely patent on follow-up venography (Figure 6b). At that time, he had returned to unrestricted right upper extremity activity and full-time work with heavy manual labor. He has since remained free of symptoms on long-term anticoagulation treatment with apixaban.

Case 2

An otherwise healthy 21-year-old male college student developed shortness of breath and chest discomfort in February 2023 and was treated with inhalers and antibiotics with no improvement. In July 2023, he had worsening shortness of breath and hemoptysis and presented to a local medical center, where a chest CTA showed bilateral PE. Lower extremity ultrasound was negative for DVT, but an upper extremity Duplex ultrasound showed left axillary-SCV thrombosis, and he was started on apixaban. 

The patient returned to the hospital in August 2023 with a chest CTA showing interval proximal extension of bilateral PE. Echocardiography showed dilation of the right atrium and ventricle, with depressed right ventricle function and RVSP of 97 mm Hg. He underwent pulmonary angiography with thrombolytic drug infusion, and on the following day, he had diminished clot burden in the left lower lobe but no change in the right lower lobe. Repeat echocardiography showed a normal right atrium and mild right ventricular dilatation with borderline function and RVSP of 81 mm Hg. Blood studies were negative for a thrombophilia disorder including antiphospholipid, factor V Leiden, and prothrombin gene mutations. The anticoagulation regimen was changed to enoxaparin, and although initially scheduled to undergo left first rib resection for VTOS, this plan was placed on hold due to ongoing pulmonary hypertension. 

**Figure 7:**
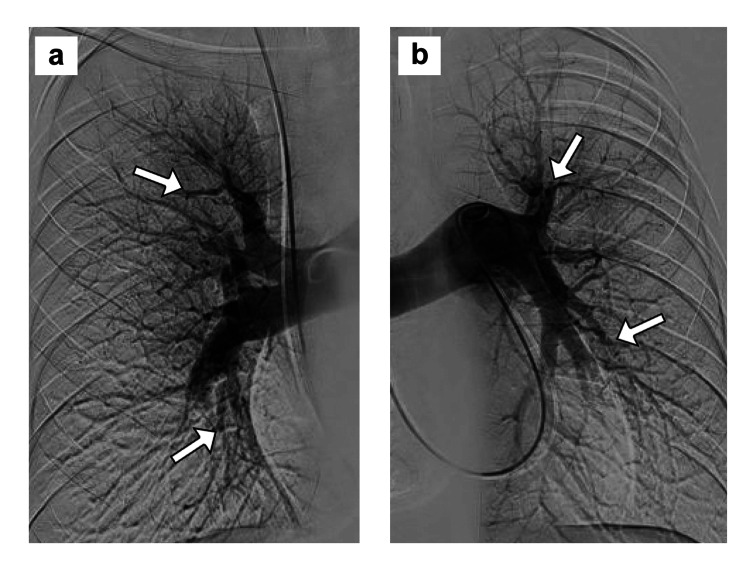
Pulmonary angiography in case 2 PA contrast injections on the a) right and b) left sides, showing multiple bilateral peripheral PA emboli (white arrows) characteristic of CTEPH. CTEPH: chronic thromboembolic pulmonary hypertension; PA: pulmonary artery

In November 2023, the patient had a follow-up echocardiogram showing moderately decreased left ventricular systolic function with an ejection fraction of 39% and grade 1 diastolic dysfunction. The right atrium was mildly dilated, the right ventricle moderately dilated, RVSP was 77 mm Hg, and there was mild tricuspid regurgitation. The patient underwent right heart catheterization, showing a right atrial pressure of 7 mm Hg, PA pressure of 82/35 (mean 56) mm Hg, and PAWP of 3 mm Hg. Pulmonary angiography showed decreased perfusion with severely diminished vessels bilaterally, right worse than left, characteristic of CTEPH (Figure 7a, 7b). He was started on riociguat and noted a moderate improvement in dyspnea over the next three weeks, but he still had dyspnea during moderate exertion (NYHA functional class II). He had no chest pain, lightheadedness, presyncope, syncope, recurrent hemoptysis, palpitations, or lower extremity edema and was not requiring supplemental oxygen. 

**Figure 8:**
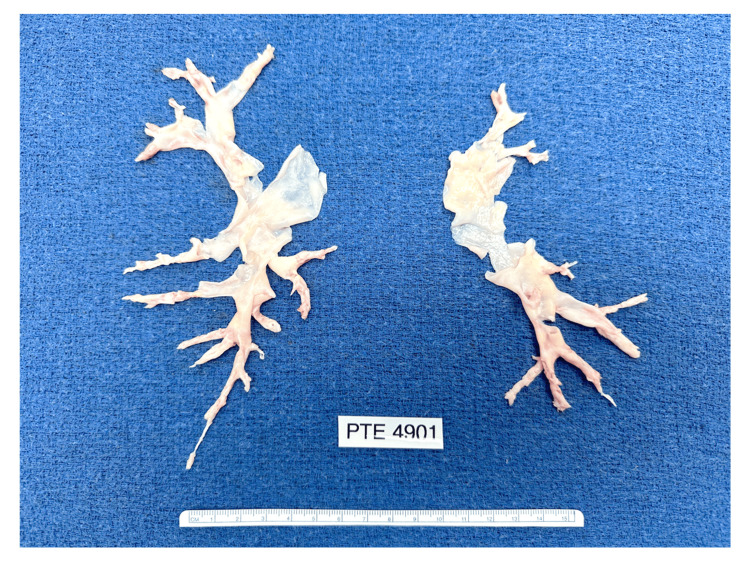
Surgical specimens in case 2 Operative photograph of specimens removed at the time of bilateral pulmonary thromboendarterectomy.

The patient was referred to an academic medical center for further assessment and surgical intervention. Pulmonary ventilation-perfusion scintigraphy showed multiple bilateral subsegmental perfusion defects. An echocardiogram showed severe right atrial dilatation, severe right ventricle enlargement with reduced systolic function, RVSP of 75 mmHg, mild-to-moderate tricuspid regurgitation, and left ventricular ejection fraction of 50%. On December 13, 2023, the patient underwent bilateral pulmonary thromboendarterectomy under deep hypothermic circulatory arrest, with an aortic cross-clamp time of 139 minutes, a cardiopulmonary bypass time of 252 minutes, and total circulatory arrest time of 54 minutes, in increments of 20 minutes or less with each arrest. There was significant bilateral thromboembolic disease with most of the disease in the segmental or subsegmental PA branches. The PA dissection plane was started more proximally in the normal but slightly thickened intima and then carried into the distal branches, thereby characterized as level III disease in the University of California San Diego classification (Figure 8). 

The patient had an uncomplicated recovery from surgery and was continued on warfarin, and within the next six weeks, he had a marked improvement in respiratory symptoms. In January 2024, the patient resumed follow-up with his pulmonary and hematology specialists. An echocardiogram showed normal right and left ventricular function (ejection fraction 53%) and RVSP of 22 mm Hg, indicating resolution of CTEPH.

The patient was subsequently seen in consultation for further assessment of VTOS. He denied having experienced upper extremity or lower extremity swelling and had no left upper extremity pain, numbness, weakness, swelling, or skin color changes despite the well-documented left axillary-SCV thrombosis. On March 28, 2024, the patient underwent successful left paraclavicular thoracic outlet decompression with resection of the anterior and middle scalene muscles, the subclavius muscle, and the entire first rib, along with external venolysis to remove fibrous scar tissue from around the axillary-SCV. An intraoperative completion venogram showed a widely patent axillary and distal SCV with no thrombus or residual stenosis and rapid filling of the innominate vein through enlarged mature collateral veins.

The patient had an uncomplicated recovery from this operation and resumed anticoagulation with warfarin, with no arm swelling or signs of venous congestion. At a follow-up visit in May 2024, Duplex ultrasound showed minimal non-occlusive chronic left SCV thrombus, much improved from the previous study prior to operation. At this point, the patient had completed postoperative physical therapy and was taking no medications beyond anticoagulation. He had resumed normal activities, including playing basketball and golf, with no arm swelling, shortness of breath, or activity intolerance. He has reported no symptoms or limitations in follow-up since that time.

## Discussion

VTOS is characterized by extrinsic compression and fibrotic stenosis of the SCV within the costoclavicular space, resulting in axillary-SCV thrombosis [10-14]. This is a form of treatable secondary/provoked DVT rather than a blood coagulation abnormality, for which catheter-based venography, mechanical thrombectomy and/or thrombolysis, and balloon angioplasty, followed by interval surgical decompression, are typically recommended [12-14]. Recent studies indicate that more than 90% of patients have successful and durable outcomes after thoracic outlet decompression, with complete first rib resection and selective SCV reconstruction based on intraoperative venography [12].

Approximately 20-25% of patients presenting with axillary-SCV thrombosis due to VTOS will have detectable PE at the time of initial symptoms, but this is likely an under-recognized occurrence [10-19]. Because VTOS is caused by high-grade fibrotic stenosis of the central SCV in the costoclavicular space and the bulk of thrombus develops in the distal axillary-SCV, the amount of thrombus that can embolize to the lung is inevitably small in volume, arising from the short proximal cul-de-sac at the junction of the SCV with the internal jugular and innominate veins. Most PE occurring in this condition are thereby minimally symptomatic and often identified as incidental findings on chest imaging [10-13]. However, patients with effort dyspnea should be fully evaluated to rule out distal CTEPH. Since these emboli are small in nature, repeated episodes are required to obstruct enough pulmonary vascular bed to result in symptoms. Younger patients may be asymptomatic at rest or with light exertion and only notice dyspnea with higher levels of exertion. 

The patients presented here were unique in having developed multiple small PE, which remained unresolved and ultimately progressed to CTEPH. Due to the chronic, scarred, and fibrotic nature of these peripheral emboli, they are not amenable to catheter-directed pulmonary thrombectomy. In fact, in patients with significant pulmonary hypertension and right ventricular strain, attempts at embolectomy can be detrimental causing hemodynamic collapse. Therefore, diagnosis of CTEPH must be ruled out in these patients prior to any planned procedure. In each case, the initial diagnosis of axillary-SCV thrombosis due to VTOS was delayed for several months with no restrictions placed on upper extremity activity, which likely resulted in repetitive thromboembolism and progressive accumulation of thrombus in the peripheral pulmonary artery branches. While each patient had ongoing anticoagulation treatment, the presence of severe respiratory failure thereby complicated the usual surgical approach to VTOS, requiring deferral of thoracic outlet decompression until resolution of the pulmonary hypertension.

The optimal screening study for CTEPH is ventilation-perfusion scintigraphy to identify persistent mismatched perfusion defects after at least three months of anticoagulation treatment [22,24]. Right heart catheterization is also necessary to demonstrate precapillary pulmonary hypertension, defined as mean PA pressure > 20 mm Hg, PAWP < 15 mm Hg, and PVR > 2 Wood units [24]. Pulmonary angiography is of value in assessing the extent and location of thromboembolic disease, particularly for surgical planning. Even in patients with CTEPH and a defined site of DVT as a source of PE, it is important to obtain full hematologic testing to assess for the possibility of an underlying thrombophilia disorder, such as antiphospholipid syndrome [4,5]. Medical management of CTEPH consists of lifelong anticoagulation. For patients who are deemed inoperable, treatment with the soluble guanylate cyclase stimulator, riociguat, is the only FDA-approved therapy for this condition. Pulmonary balloon angioplasty has recently emerged as an additional and potentially effective intervention for disease located in the more distal pulmonary vessels, applicable to patients unsuitable for surgical treatment [22,24,25].

Pulmonary thromboendarterectomy is well-established as a successful and potentially curable approach to CTEPH, with up to 85-90% of patients determined to be operable [6,7,21,22]. Perioperative mortality rates are approximately 2% at experienced surgical centers, and advances in surgical technique have allowed patients with more distal disease, extending into the segmental and subsegmental PA branches, to be successfully treated. The long-term outcomes after pulmonary thromboendarterectomy indicate improvements in quality of life, function, and exercise capacity compared to patients treated non-operatively [21,22]. It is worth emphasizing that these operations are exceptionally challenging and that optimal results are obtained primarily in experienced centers in the context of a team dedicated to multidisciplinary management of pulmonary vascular disease.

The patients presented here serve to highlight the potential role of VTOS in development of CTEPH and emphasize that SCV thrombosis should be suspected in young active patients presenting with CTEPH and no other identified source of DVT. Upper extremity Duplex ultrasound is useful for initial assessment as it can easily detect thrombus that has propagated retrograde into the lateral SCV and axillary vein, but in many patients with VTOS, the thrombus is confined to a localized area of the central SCV. It is important to recognize that Duplex ultrasound studies have a significant false-negative rate in VTOS (approximately 20%), as it is often difficult to accurately image the central SCV behind the clavicle [26]. Patients with suspected VTOS and negative upper extremity ultrasound should thereby undergo contrast-enhanced cross-sectional imaging (e.g., CTA) or catheter-directed venography to definitively evaluate the SCV. Venography has the added advantage that it allows immediate catheter-directed intervention by mechanical thrombectomy or thrombolysis and possible SCV balloon angioplasty. This principle can be extended to patients with PE and/or CTEPH and no other identified source of DVT.

There are several previous publications that include patients with the combination of CTEPH and VTOS. While some of these patients did undergo surgical treatment for each condition, this is not often described and these reports include only limited follow-up information [27-30]. The present report thereby extends these published observations to describe some of the first patients to undergo a successful staged surgical approach to CTEPH in combination with VTOS, in a manner that definitively addresses both problems in a timely sequence.

## Conclusions

We present two novel clinical cases to highlight the potential for SCV thrombosis associated with VTOS to cause PE and CTEPH, indicating that upper extremity DVT should be considered in patients with PE for which no other source of venous thrombosis has been identified. We also emphasize that PE can occur in patients with VTOS and in rare situations even lead to the development of CTEPH and right ventricular dysfunction. The patients presented here are some of the first to have successful staged surgical treatment for CTEPH caused by SCV thrombosis due to VTOS. They illustrate the value of comprehensive multidisciplinary management for this complicated combination of rare conditions, involving a team of cardiologists, pulmonologists, hematologists, interventional radiologists, and cardiac and vascular surgeons, to achieve an excellent clinical outcome.
